# Enhanced Mechanical Property of Polyamide-6/Graphite Sheet Composites with Segregated 3D Network Binary Structure for High Thermal Conductivity

**DOI:** 10.3390/polym15041041

**Published:** 2023-02-19

**Authors:** Yao Gao, Yong Li, Xiangwei Kong, Meng Ma

**Affiliations:** 1 College of Optoelectronic Manufacturing, Zhejiang Industry & Trade Vocational College, Wenzhou 325000, China; 2 College of Materials Science and Engineering, Zhejiang University of Technology, Hangzhou 310014, China

**Keywords:** microspheres, percolation threshold, segregated structure, mechanical properties

## Abstract

Segregated conductive polymer composites exhibit excellent electrical properties with a low percolation threshold. However, the mechanical properties of the segregated conductive polymer composites were always poor because the conductive fillers at the interfaces hinder polymer chain diffusion and thus lead to weak interfacial interaction between the conductive fillers and the polymer matrix. In this paper, polyamide-6 and polyamide-612 microspheres were synthesized via the in situ anionic ring opening of caprolactam and laurolactam. Segregated graphite sheets/polyamide-6(GS/PA6) and polyamide-612(PA612) composites with good mechanical properties were realized via high-pressure solid-phase compression molding. The microstructures of the composite samples were observed by scanning electron microscopy, which showed that the formation of a GS-conductive network at the PA6 granule interfaces in the segregated conductive structures and the adopting of PA612 considerably improved the interfacial adhesion of the composites. A superior impact strength of 5.1 kJ/m^2^ was achieved with 50 wt% PA612 loading owing to improvements in the interface compatibility between PA6 and GS. The composites possessed an ultralow percolation threshold, which was ascribed to the segregated network structure being successfully constructed inside the material. As for GS/PA6 composites, the combination of segregated GS-conductive networks achieved an ultralow percolation of 2.8 vol%. The percolation of 80PA6/20PA612-GS composites was slightly higher, measuring up to 3.2 vol%. Moreover, the thermal conductivity of the 80PA6/20PA612-GS composites increased from 0.26 to around 0.5 W/(m·K), which was 1.9 times larger than the pure polyamide.

## 1. Introduction

Over the past few decades, preparing conductive polymer composites with a segregated structure has proved to be an effective method to improve their electrical properties, generating significant academic and industrial interest [1,2]. At present, segregated conductive polymer composites have shown great application value in the fields of sensors [3,4], antistatic materials [5,6] and electromagnetic shielding [7,8,9,10]. As for normal conductive polymer composites (CPCs), numerous conductive fillers are needed to obtain a satisfactory conductive performance, which will require complex processing and exhibit inferior mechanical properties, limiting the application of CPCs [1]. The conductive fillers of segregated CPCs (s-CPCs) are selectively segregated at the interfaces of polymeric granules instead of being randomly distributed throughout the conductive polymer composites, which is beneficial when constructing conductive networks, providing more-efficient conductive paths to considerably reduce the percolation threshold and improve the electrical and thermal conductivities thanks to the higher local concentration of conductive fillers [11,12,13,14,15,16]. Since Turner et al. [17,18] first proposed the segregated structure of conductive polymer composites, many polymers have been used to prepare s-CPCs, such as polymethyl methacrylate (PMMA) [19], polystyrene (PS) [20,21], ultrahigh-molecular-weight polyethylene (UHMWPE) [22,23] and other polymers [24,25,26,27,28,29].

However, the isolation structure of s-CPCs makes the conductive filler dispersed at the polymer interface, which prevents the diffusion of the polymer molecular chain. In addition, the selective dispersion of the fillers results in a significant increase in the density at the interface, thus resulting in the porous microstructures in CPCs. Therefore, the mechanical properties of the segregated composites are extremely poor, owing to interfacial phase separation and the poor compatibility between the polymer matrix and fillers [12]. The practical application of segregated CPCs as a kind of important functionalized polymer material has been limited to low. Therefore, improving the mechanical properties of segregated conductive polymer composites is of great significance for expanding their applications, and many studies have been performed to achieve this goal. For example, Yu et al. [30] obtained largely enhanced mechanical properties for segregated carbon nanotube/poly(vinylidene fluoride) (PVDF) composites by using solid-phase extrusion (SPE). They found that a mechanically interlocked state between the PVDF and carbon nanotubes (CNTs) improved the interfacial bonding by producing a large number of oriented crystals, which resisted mechanical failure, leading to improvements in the strength and fracture toughness of the segregated CNTs/PVDF composites. Cui et al. used small CB nanoparticles to decrease the gap between the polymer matrix (UHMWPE) and the large graphite flakes so that the mechanical properties would be enhanced [31]. Yan et al. prepared a high-performance electromagnetic interference-shielding composite via high-pressure solid-phase compression molding [18]. They believed that the enhanced mechanical properties could be considered as the mutual diffusion of PS chains under high pressure across the interface between PS and rGO. Cui et al. fabricated a segregated structure in a typical low-melt-viscosity polymer (i.e., PLA), making use of the melting temperature (Tm) discrepancy between homocrystallites (HCs) (160–180 °C) and stereocomplex crystallites (SCs) (∼220 °C) [32]. Their results showed a superior electrical conductivity of 12 S/m with only 1 wt% CNTs. Shi et al. [33] prepared poly(-l-lactide)/polyε-caprolactone)/multiwall carbon nanotube (PLLA/PCL/MWCNT) composites with an electrically conductive segregated structure, and the Young’s modulus of the segregated CPCs was 10% higher than that of the corresponding conventional composites. Zhang et al. [26] reported a facile and ecofriendly method to develop CNTs/polypropylene (PP) segregated composites with simultaneously improved mechanical performance using two commercially available copolymerization polypropylenes, including high-melting PP and low-melting PP. The elongation at the break of the segregated CNT/PP composite was four times higher than the conventionally injection-molded samples. Even today, it remains a considerable challenge to enhance the mechanical properties of segregated composites.

Polyamide, as an important engineering plastic, has excellent mechanical properties, high wear resistance and chemical resistance, and it is applied in electronic communications [34], the automotive industry [35], biological engineering [36,37] and other fields [11]. When applied in the field of electronic appliances, most segregated CPC (s-CPC) matrices possess a high-melt viscosity to resist plastic deformation and prevent the conductive fillers from permeating into the polymer matrix during processing, thus facilitating the formation of a segregated conductive network along the boundaries of polymer particles. UHMWPE, PS and PMMA are used mainly as polymer matrices in s-CPCs. The conductive filler’s diffusion into polyamide particles is a big problem during hot compacting when creating segregated CPCs; thus, research on the preparation of s-CPCs based on polyamides is limited owing to its low high-melt viscosity. In this work, the PA6 microspheres and PA612 microspheres were first prepared via the anionic ring-opening polymerization according to the phase-inversion morphology generated in the PS/PA6 and PS/PA612 blends. Segregated GS/PA6 and PA612 composites were prepared by high-pressure solid-phase compression molding. For segregated polyamide-6 and graphite sheet composites, the mechanical properties were relatively poor because of the separation between the two phases. In order to overcome this defect, polyamide-612 was added as a binder to improve the mechanical properties. We studied the electrical, mechanical and microstructure properties and the thermal conductivity of the resulting GS-based composites. Herein, we report a novel and facile method to develop polyamide-segregated CPCs with improved mechanical properties; furthermore, the fabrication process in this work can be applied in other polymer matrices by adding similar polymers with different melting points.

## 2. Materials and Methods

### 2.1. Materials

Caprolactam (CL) and laurolactam (LL) were kindly supplied by BASF. A graphite sheet with a purity above 95% was purchased from Qindao Guangli Co., Ltd. (Qindao, China). Commercial-grade PS (PG-22) and 2,4-toluene diisocyanate (TDI, analysis purity) were obtained from Taiwan Qimei Co., Ltd. (Taiwan, China) and Alfa Aesar (China) Chemical Co., Ltd. (Shanghai, China), respectively. Sodium hydroxide (NaOH; analysis purity) was provided by Xilong Chemical Co., Ltd. (Shantou, China) Toluene (analysis purity) was purchased from the Shanghai Chemical Reagents Company (Shanghai, China). All the reagents and solvents were of analytical grade and were used without further purification.

### 2.2. Preparation of Polyamide Microspheres and Composites

**Figure 1:**
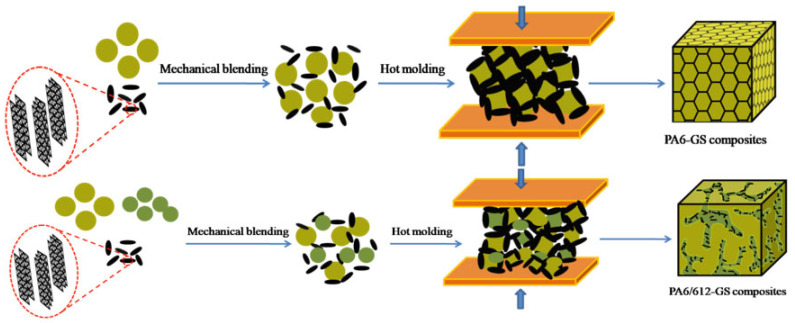
Schematic of the fabrication of the segregated PA6 -GS composite and PA6/612-GS composite under high pressure.

Polyamide microspheres were synthesized via the in situ anionic ring opening of caprolactam and laurolactam [38,39,40]. The fabrication process of polyamide/graphite sheet composites is schematically illustrated in Figure 1. The process included mainly three main steps: the synthesis of polyamide microspheres (Step 1), the mechanical blending of the graphite sheet with PA6 and PA612 particles (Step 2) and hot molding (Step 3).

Initially, polyamide microspheres were prepared according to the phase-inversion morphology generated in the PS/PA blends. A certain amount of polystyrene (PS) was mixed with caprolactam (CL) at 100 °C for 5 h to obtain a homogeneous transparent mixture. Water in the reaction system was removed by distilling it under reduced pressure. Anionic polymerization was initiated by NaOH and TDI, and the mixture was then immediately poured into a mold preheated to 170 °C and polymerized in an oven at 170 °C for 30 min. Finally, the obtained PA6/PS blends were shattered and then immersed in toluene for 10 h to dissolve the PS phase. The product was washed by distilled water to remove CL monomer and dried at 70 °C in a vacuum oven for 12 h. The PA microspheres were finally obtained. The PA612 microspheres were similarly synthesized via the in situ anionic ring opening of caprolactam and laurolactam. Additionally, the graphite sheet was mixed with PA6 and PA612 particles by high-speed mixer for 5 min. The mixture was heated by the vacuum drying oven at 50 °C for 2 h. Finally, the GS-coated PA6 and PA612 particles were consolidated into the cylindrical billet at 200 °C for 8 min under a pressure of 10 MPa. As a comparison, PA6-GS composites were prepared by the same method. For subsequent analysis and characterization, the prepared composites were coded PA6/612-GS(x/y-z), where x, y and z indicated the percentages of PA6, PA612 and GS, respectively.

### 2.3. Characterization

The synthesized polyamide microspheres were ultrasonically dispersed in ethanol (1 g/mL) and dropped on the slide. After drying, all samples were gold plated under vacuum, and thereafter, their morphologies were observed by scanning electron microscopy (SEM; Vega ΙΙ XMU instrument, Tescan, Czech Republic) at an operating voltage of 15 kV. For the segregated structure investigation, the cross-sectional morphology of the samples was also observed by using SEM at an acceleration voltage of 15 kV.

The melting temperature (Tm) of the synthesized polyamide was characterized by a differential scanning calorimeter (TA instruments, DSC Q100, New Castle, DE, USA), with nitrogen as the protective atmosphere. The mass of each sample was about 5 to 6 mg, and the samples were dried in a vacuum oven at 80 °C for 12 h before the test. The sample was first heated from normal temperature to 250 °C at a heating rate of 10 °C/min and maintained at 250 °C for 3 min to erase the thermal history. Next, it was cooled down to 30 °C at a cooling rate of 10 °C/min and kept constant for 3 min. Finally, the temperature increased to 250 °C at a rate of 10 °C/min.

The volume conductivities of the composite samples with conductivities higher than 10^−6^ S m^−1^ were carried out using a 4-probe method by the RTS resistivity measurement system (RTS-8, Guangzhou, China), and the volume of electrical conductivities below 10^−6^ S m^−1^ were measured by using a high-insulation-resistance meter (ZC-90F, Shanghai Sute Electric Co. Ltd., Shanghai, China). The thermal conductivities were measured by using a thermal conductivity tester (DRP-II, Xiangtan, China).

Tensile tests to determine the tensile strength, modulus and elongation-at-break values were carried out by using a universal testing machine (Instron 5966, Instron Corporation, Canton, OH, USA) with a load cell of 10 kN and a crosshead deformation rate of 5 mm/min at room temperature, according to the ASTM D638-22 (standard test method for tensile properties of plastics) standard. Furthermore, the notched impact test specimens, having dimensions of approximately 80 × 10 × 2 mm were compression molded (L × W × B) according to the ISO 179-2 (the plastics determination of Charpy impact properties) standard. All the corresponding presented data were calculated as the average and the standard deviation of at least five specimens for each composition.

## 3. Results and Discussion

### 3.1. Characterization of PA6, PA612, and Graphite Sheet

**Figure 2:**
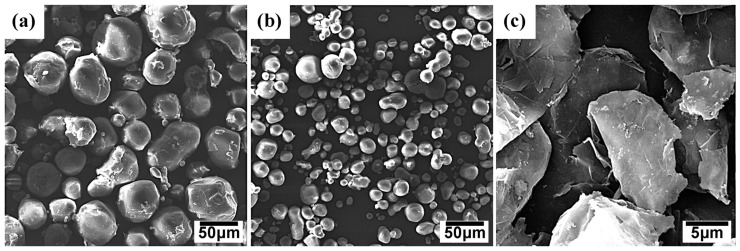
SEM micrographs of PA6 microspheres (**a**), PA612 microspheres (**b**) and graphite sheets (**c**).

As mentioned above, PA6 and PA612 microspheres were synthesized via the in situ anionic ring opening. By etching the PS matrix with solvent, the PA6 and PA612 spheres were easily obtained. The size of the PA6 and PA612 microspheres was verified by the SEM displayed in Figure 2. PA6 is uniform, with a diameter about 50 μm, while PA612 is around 20 μm. The different sizes are beneficial for helping the composites form a dense structure. As shown in Figure 2c, the graphite sheet is approximately 10 μm, which is much smaller than the size of the PA6 microspheres and PA612 microsphere, which could fill the gap between them to further densify the composite.

**Figure 3:**
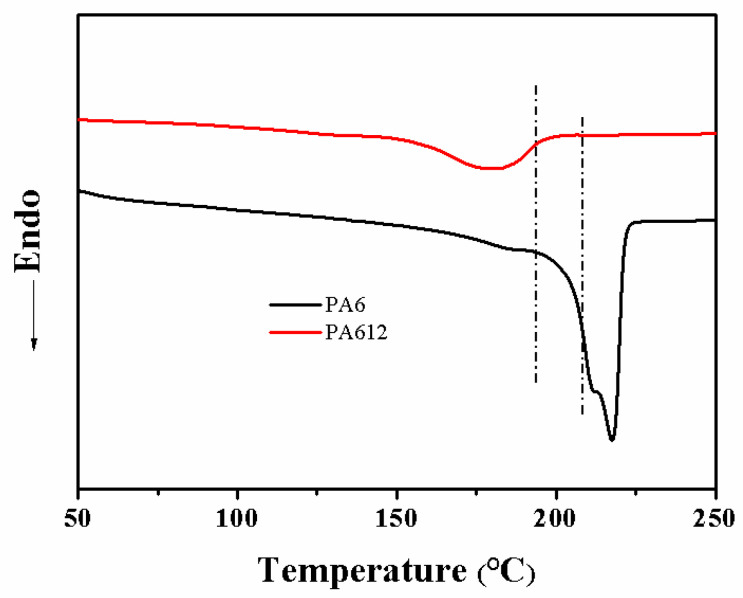
DSC curves (appropriate compression temperature for the formation of a segregated structure, marked) for PA6 microspheres and PA612 microspheres.

When forming the segregated structure, it is important to control the processing temperature. If the temperature is too high to decrease the polymer’s viscosity, the fillers easily infiltrate into the inner part of the polymer matrix, resulting in the formation of a poorly segregated structure. On the contrary, the powder cannot completely melt if the processing temperature is too low, leading to an undesired morphology, resulting in the poor interface adhesion or even the hole between the polymer and the fillers. To obtain the proper processing temperature, the different melting points Tm of the PA6 and PA612 microspheres were investigated by DSC. As shown in Figure 3, PA612 has only one melting point, while PA6 has two melting points. The melting point of the PA612 microsphere (Figure 3) is about 180 °C, while the melting points of the PA6 microsphere are about 210 °C and 220 °C. The Tm (180 °C) of PA612 is lower than both the Tms of PA6 (210 °C and 220 °C). To ensure that PA6 maintains a solid phase while keeping PA612 in a melting state, the appropriate processing temperature range, as marked by the line in Figure 3, needs to be selected. In this study, a temperature of 200 °C is chosen as the hot processing temperature; thus, composite processing is considered to be a solid-phase compression molding. In addition, the composites not only can contain isolation structures but also have good interface adhesion, according to the compatibility of PA612. Therefore, the mechanical properties of the polyamide-segregated composites can be improved.

### 3.2. The Electrical Conductivity of PA6/612-GS Composite

**Figure 4:**
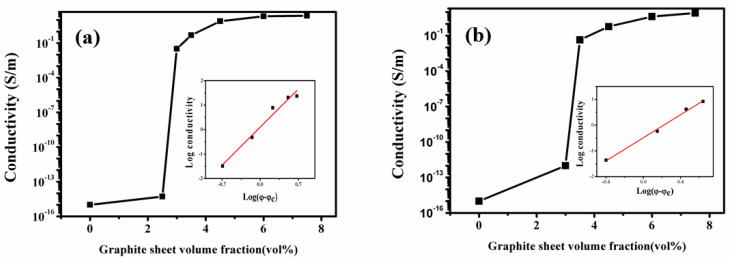
Electrical conductivity of the PA6-GS composites (**a**) and 80PA6/20PA612-GS composites (**b**) as a function of GS content.

The GS content’s dependence on electrical conductivities for the PA6-GS and PA6/612-GS segregated composites is illustrated in Figure 4. In both cases, with the increase in GS concentration, the electrical conductivities of the PA6-GS and PA6/612-GS composites substantially increase with GS loading and exhibit the characteristic percolative behavior. For the PA6-GS composite, the electrical conductivity increases by 14 orders of magnitude in a very narrow GS content range, from 0 to 2.8 vol%. However, for the PA6/612-GS composite, the percolation value slightly increases to 3.2 vol% because the GS enters the molten PA612 during processing. However, both composites possess an ultralow percolation threshold, which should be ascribed to the segregated network structure constructed inside the material.

**Figure 5:**
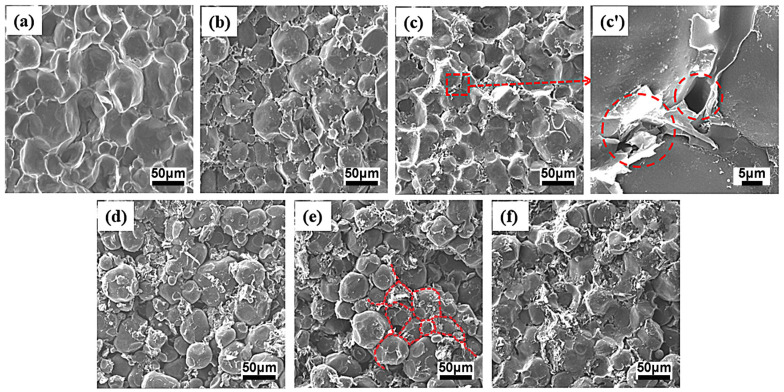
SEM images of PA6-GS composites of different GS contents: 0 wt% (**a**), 1 wt% (**b**), 3 wt% (**c**), 5 wt% (**d**), 7 wt% (**e**), and 9 wt% (**f**). A higher magnification of selected sections of part (**c**) is shown in part (**c’**).

**Figure 6:**
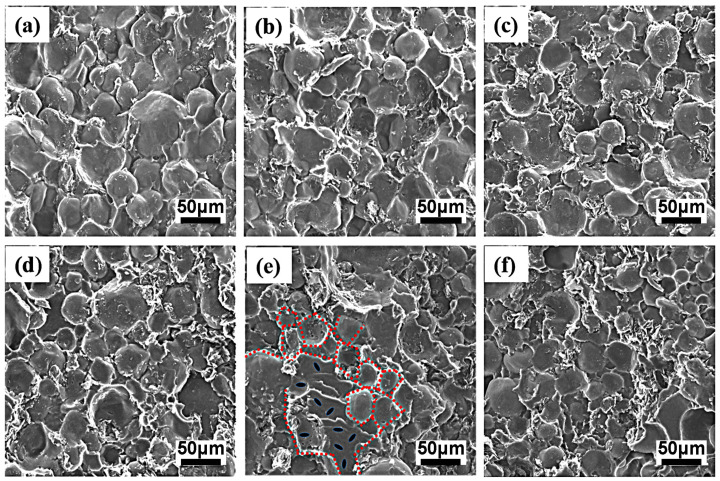
SEM images of 80PA6/20PA612-GS composites of different GS contents: 0 wt% (**a**), 1 wt% (**b**), 3 wt% (**c**), 5 wt% (**d**), 7 wt% (**e**), and 9 wt% (**f**).

To provide insights into the structural formation and the evolution of conductive networks, the scanning electron microscopy images of the PA6-GS and 80PA6/20PA612-GS composites with various GS contents are shown in Figure 5 and Figure 6, respectively. The typical segregated network structures of the PA6-GS composites are shown in Figure 5e, and some PA6 matrix particles showed plastic deformation under high pressure during processing. Figure 5a–f shows that the PA6 matrix becomes rougher, while the interface of the PA6 matrix becomes clearer with the increase in the content of GS, which is because the incompatible GS distributes mainly at the interfaces of the PA6 particles (Figure 5e). Regardless of the GS loading, a selective distribution of GS around the PA6 domains can be observed, which means that the graphite sheets were restricted from penetrating into the PA6 granules’ interiors during processing. However, some microvoids exist as interfacial defects, indicating the poor interfacial adhesion of the PA6-GS composites at higher magnification (Figure 5c′). Owing to the high diffusion barrier, the PA6 chains could not fully fill the enriched GS layer gaps via conventional compression molding. Therefore, we introduced the PA612 granules, which were molten at the processing temperature, into the composites that were used as binders to improve the connection between the PA6 matrix and the GS filler. Figure 6 also shows the composites’ typical segregated network structures, where the PA6 matrix is becoming rougher with the increasing GS content. Meanwhile, there is no visible defect along the segregated conductive path, indicating an improved interfacial adhesion, which would have a significant impact on improving mechanical properties, as described below. Moreover, GS stacking in the 80PA6/20PA612-GS composites could be more compact and ordered, which would facilitate the formation of an effective thermal conductivity network, as shown in Figure 6e.

### 3.3. The Thermal Conductivity of PA6/612-GS Composite

**Figure 7:**
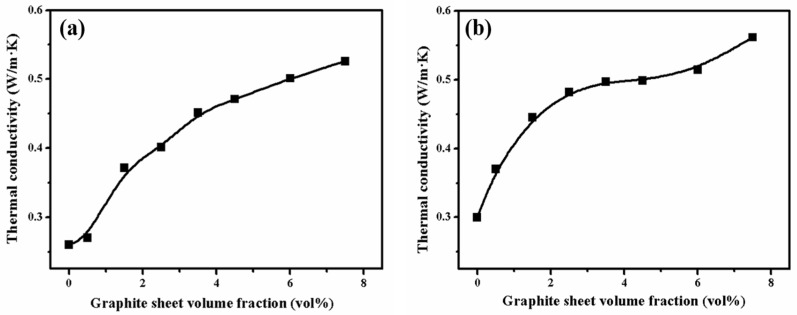
Thermal conductivity of the PA6 -GS composites (**a**) and 80PA6/20PA612-GS composites (**b**) as a function of GS contents.

Because of the perfect thermal conductivity of graphite sheets, the existence of a conductive network could obviously improve the composites’ thermal conductivities [41]. The dependence of the GS content on thermal conductivity for the PA6-GS and 80PA6/20PA612-GS composites are presented in Figure 7. The thermal conductivity of the PA6-GS and 80PA6/20PA612-GS composites increased in the wake of the increasing GS content. The thermal conductivity of the latter was invariably higher than those of the former with the same composition. In particular, the thermal conductivity is considerably increased, up to 0.5 W·m^−1^·K^−1^, with only 5 vol% GS loading. This implies that the graphite sheets in the 80PA6/20PA612-GS composites are more efficient in forming thermal conductive networks than the PA6-GS composites are. On the basis of discussions about SEM results and electrical properties, we concluded that the 80PA6/20PA612-GS composites can make the stacking of the GS more compact, with good interface between the PA6 matrix and GS, consequently giving rise to the superior thermal conductivity in the 80PA6/20PA612-GS composites.

### 3.4. The Mechanical Properties of PA6/612-GS Composite

**Figure 8:**
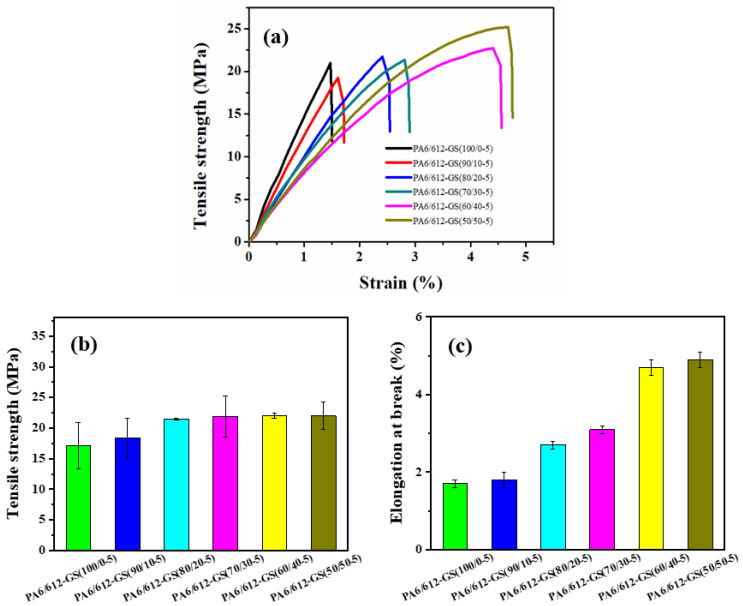
Mechanical properties of PA6/612-GS composites: (**a**) stress–strain curves, (**b**) tensile strength, and (**c**) elongation at break.

In practical applications, mechanical properties are one of the most considerable characteristics of materials [42], and they are a major limitation for the engineering applications of segregated CPCs. Thus, it is of great significance to improve the mechanical properties of segregated CPCs [43,44,45]. To evaluate the potential applications for the composites, the mechanical performances of the PA6/612-GS composites were explored by using a tensile test. The representative mechanical property data, including the tensile strength, Young’s modulus, and elongation at the break of the PA6/612-GS composites, are summarized in Table 1. Moreover, the stress–strain curves of the PA6/612-GS composites obtained from the tensile test are shown in Figure 8a, and the tensile strength and elongation at the break of the PA6/612-GS composites with various PA612 contents are also presented in Figure 8 for comparison.

The typical stress–strain curves of the PA6/612-GS composites shown in Figure 8 illustrate the relatively ductile fracture for the PA6/612-GS composites compared with the brittle failure for the PA6-GS composites. The PA6-GS(100-5) composite exhibited brittle failure at around 1.7% strain. In contrast, the addition of PA612 could significantly improve the elongation at the break of the composites. As shown in Figure 8b,c, for the PA6/612-GS composites, with the increase in the PA612 content, both the tensile strength and the elongation at the break of the PA6/612-GS composites showed a gradual increase. The average tensile strength and elongation at the break were improved to 21.5 MPa and 2.7%, respectively, with the addition of 20 wt% PA612, showing a 20% and a 58% increase compared with the PA6-GS(100-5) composite. When PA612 content increased to 50 wt%, the tensile strength was about 22.0 MPa and the elongation at break rose to 4.9%, which increased by 188% compared with that of PA6-GS(100-5) composite, indicating that the incorporation of the PA612 into PA6-GS composite was effective in improving toughness. As we know, the solid PA6 granules were poorly compatible with GS. For the PA6-GS composite, the poor interfacial adhesion caused premature debonding, which led to an early propagation of the void and brittle failure. The improved mechanical properties of the PA6/612-GS composites could be proved from the SEM above.

**Figure 9:**
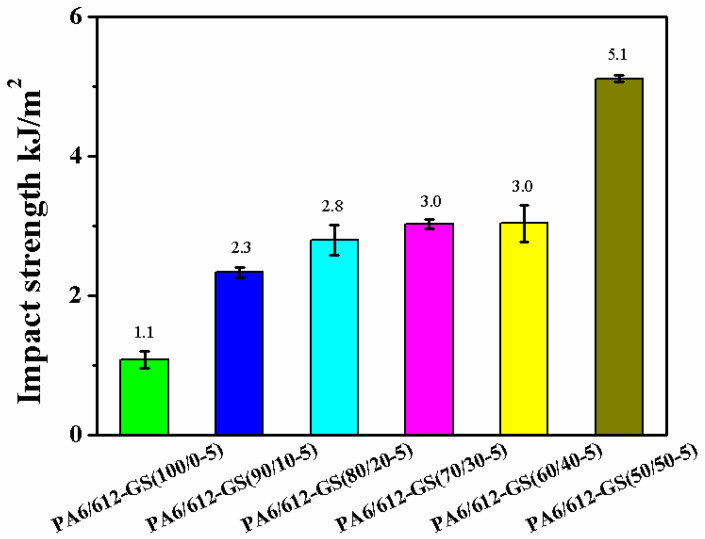
Impact strength of PA6/612-GS composites: average values of resistance to impact, obtained after five tests.

To further investigate the mechanical properties of the PA6/612-GS composites, we also evaluated the impact properties of the samples by using the Charpy unnotched impact test, as shown in Figure 9. Five tests were carried out, and the average of the obtained values are shown in the bar graph of Figure 9. The PA6-GS composite was extremely brittle, with an impact strength of 1.1 kJ/m^2^. The impact strength of the PA6/612-GS composite gradually increased with the increasing PA612 content. The impact strength of the PA6/612-GS composites with 10 wt% PA612 was 2.3 kJ/m^2^. There was a considerable increase in toughness by adding 10 wt% PA612. When the PA612 reached 20 wt% and 30 wt%, the impact strength of the PA6/612-GS composites increased to 2.8 kJ/m^2^ and 3.0 kJ/m^2^, respectively, which were 155% and 173% higher than the 1.1 kJ/m^2^ of the PA6-GS composite with weak interfacial adhesion, respectively. One can easily find that a remarkable improvement in the toughness of the PA6/612-GS composite is achieved by loading 50 wt% PA612. The impact strength of the PA6/612-GS composite is improved from 1.1 kJ/m^2^ to 5.1kJ/m^2^ (a 364% increase with the PA612 loading variation from 0 wt% to 50 wt%). Obviously, adopting PA612 is favorable for improving the impact property of the composite as a result of compatibilization, as shown in Figure 5 and Figure 6.

## 4. Conclusions

In summary, segregated PA6/612-GS composites were fabricated by the mechanical blending and molding process. PA6 microspheres and PA612 microspheres were successively prepared via an in situ anionic ring opening. Because the T_m_ of PA6 is much higher than that of PA612, PA6 granules could be easily reserved when an appropriate temperature was chosen for compression molding. At the same time, the segregated structure was well maintained, making the composites exhibit a low percolation threshold and excellent electrical and thermal conductivities. As for PA6/GS composites, the combination of a segregated GS-conductive network achieved an ultralow percolation, of 2.8 vol%. The percolation of the 80PA6/20PA612-GS composites was slightly up, to 3.2 vol%. Moreover, the stackings of the GS were more compact and ordered in the 80PA6/20PA612-GS composites, resulting in the superior thermal conductivity of the composites, which was 1.9 times higher than that of the pure polyamide. During the hot processing, the melting PA612 acted as the adhesive, which improved the mechanical properties of the segregated CPCs. The increase in the content of PA612, up to 50 wt%, led to an increase in the maximum elongation at break and an increase in the impact strength values of the segregated PA6/612-GS composites by 188% and 364%, respectively, compared with the PA6-GS(100-5) composite. A new way to enhance the mechanical properties of the polyamide-segregated CPCs is provided by adding similar polymers with different melting points. Further investigations to improve the mechanical properties of polyamide-segregated CPCs are necessary.

## Figures and Tables

**Table 1:** Data on typical mechanical properties of PA6/612-GS composites.

Sample	Tensile Strength (MPa)	Young’s Modulus (MPa)	Elongation at Break (%)
PA6/612-GS (100/0-5)	17.1 ± 3.8	1552.9 ± 376.3	1.7 ± 0.1
PA6/612-GS (90/10-5)	18.4 ± 3.2	1362.9 ± 180.9	1.8 ± 0.2
PA6/612-GS (80/20-5)	21.5 ± 0.1	1181.3 ± 37.4	2.7 ± 0.1
PA6/612-GS (70/30-5)	21.9 ± 3.4	1123.2 ± 73.3	3.1 ± 0.1
PA6/612-GS (60/40-5)	22.0 ± 0.4	950.5 ± 21.2	4.7 ± 0.2
PA6/612-GS (50/50-5)	22.0 ± 2.2	888.4 ± 69.1	4.9 ± 0.2

## Data Availability

Data are contained within the article.
